# Transcriptome Analysis of Sucrose Metabolism during Bulb Swelling and Development in Onion (*Allium cepa* L.)

**DOI:** 10.3389/fpls.2016.01425

**Published:** 2016-09-22

**Authors:** Chunsha Zhang, Hongwei Zhang, Zongxiang Zhan, Bingjiang Liu, Zhentai Chen, Yi Liang

**Affiliations:** ^1^Beijing Vegetable Research Center, Beijing Academy of Agriculture and Forestry Sciences/Key Laboratory of Biology and Genetic Improvement of Horticultural Crops (North China)Beijing, China; ^2^National Key Laboratory of Crop Genetic Improvement, College of Plant Science and Technology, Huazhong Agricultural UniversityWuhan, China; ^3^Key Laboratory for Biology of Greenhouse Vegetable of Shandong Province, Institute of Vegetables and Flowers, Shandong Academy of Agricultural SciencesJinan, China; ^4^Vegetable Research Laboratory, Institute of Agricultural Sciences of Xuhuai Region in JiangsuLianyungang, China

**Keywords:** *Allium cepa* L., bulb swelling, RNA-seq, sucrose metabolism, gene expression

## Abstract

*Allium cepa* L. is a widely cultivated and economically significant vegetable crop worldwide, with beneficial dietary and health-related properties, but its sucrose metabolism is still poorly understood. To analyze sucrose metabolism during bulb swelling, and the development of sweet taste in onion, a global transcriptome profile of onion bulbs was undertaken at three different developmental stages, using RNA-seq. A total of 79,376 unigenes, with a mean length of 678 bp, was obtained. In total, 7% of annotated Clusters of Orthologous Groups (COG) were involved in carbohydrate transport and metabolism. In the Kyoto Encyclopedia of Genes and Genomes (KEGG) database, “starch and sucrose metabolism” (147, 2.40%) constituted the primary metabolism pathway in the integrated library. The expression of sucrose transporter genes was greatest during the early-swelling stage, suggesting that sucrose transporters (SUTs) participated in sucrose metabolism mainly at an early stage of bulb development. A gene-expression analysis of the key enzymes of sucrose metabolism suggested that sucrose synthase, cell wall invertase, and invertase were all likely to participate in the hydrolysis of sucrose, generating glucose, and fructose. In addition, trehalose was hydrolyzed to two molecules of glucose by trehalase. From 15 to 40 days after swelling (DAS), both the glucose and fructose contents of bulbs increased, whereas the sucrose content decreased. The growth rate between 15 and 30 DAS was slower than that between 30 and 40 DAS, suggesting that the latter was a period of rapid expansion. The dataset generated by our transcriptome profiling will provide valuable information for further research.

## Introduction

Onion (*Allium cepa* L.), a group of monocotyledonous biennial herbs belonging to the Alliaceae family, is the most economically important vegetable plant (Jakše and Bohanec, 2003). It may have been the earliest cultivated form of any vegetable crop. Dating back 5000 years, onions were already an important food source in ancient Egypt. With many health-related benefits, onions are frequently recognized as having an important dietary role, especially in preventing cardiovascular disease and cancer (Havey et al., 2004). Onions can be classified as sweet or non-sweet. Their significance in cooking is determined by their taste characteristics (pungent and sweet) and flavor profile. About 80% of onion bulb dry matter consists of non-structural carbohydrates (Darbyshire and Henry, 1981). The main carbohydrate components are glucose, fructose, sucrose, and fructo-oligosaccharides. Glucose, fructose, and sucrose account for 65% of the dry matter content, which varies from ~5% of fresh weight in sweet onions to ~30% in dehydrated varieties (Darbyshire and Henry, 1979; McCallum et al., 2006).

Onion pungency is caused by a range of sulfur compounds. When onions are first cut, some of these compounds affect the eyes and produce tears (Tewari and Bandyopadhyay, 1975). A high degree of pungency can mask a high level of sugar, resulting in the onion not being considered to be sweet. Also, onions with low pungency and low sugar content can be regarded as bland. Ideally, a sweet onion will have a high sugar level and low pungency. Thus, the balance between the pungency and sugar levels determines the perception of sweetness in an onion. In all cases, the sweetness and pungency that are produced are important aspects of the formation and development of onion bulbs. Despite the literature on sucrose metabolism in plants, the genetic mechanisms involved in the formation and development of onion bulbs have not been reported.

**Figure 1:**
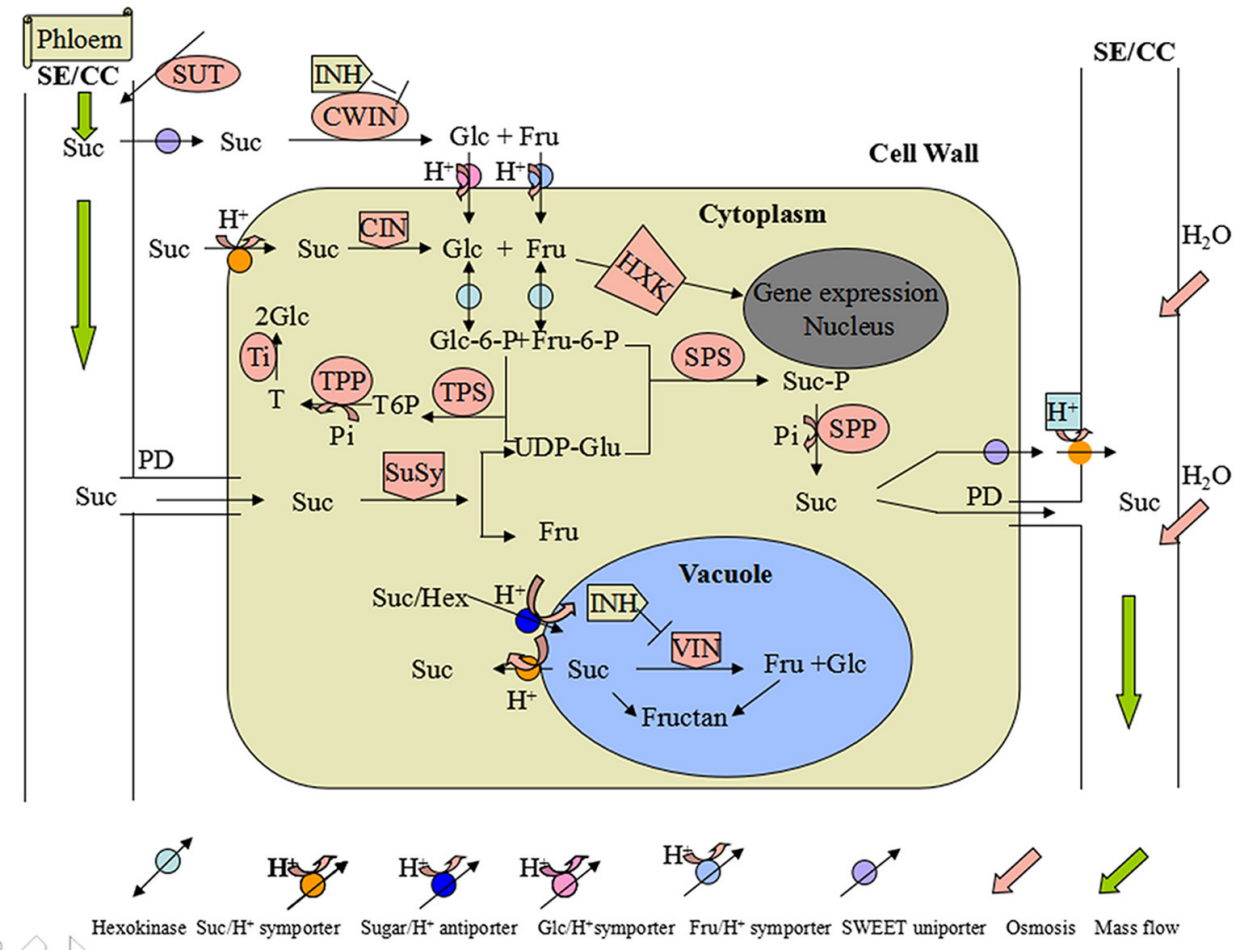
**Sucrose (Suc) loading, transport and unloading pathways in a non-photosynthetic cell**. SE/CC, sieve element/companion cell; PD, plasmodesmata; Suc, sucrose; Glc, glucose; Fru, fructose; SUT, sucrose transporter; INH, inhibitor; CWIN, cell wall invertase; CIN, cytoplasmic invertase; SPS, sucrose-phosphate synthase; Ti, trehalase; TPP, trehalose-6-phosphate phosphatase; TPS, trehalose-6-phosphate synthase; SuSy, sucrose synthase; HXK, hexokinase; VIN, vacuolar invertase.

The formation and development of onion bulbs are closely related to sucrose metabolism (Sinclair et al., 1995; Mallor et al., 2011). In the non-photosynthetic cells of higher plants, sucrose is transported from the photosynthetic apparatus and cleaved to its constituent monosaccharides, hexoses (Hexs) or phosphorylated Hexs, which can then be used either in catabolic or biosynthetic reactions (Ruan, 2014). The only known enzymatic processes of sucrose (Suc) cleavage in plants are catalyzed by invertases [Suc combines with H_2_O to generate glucose (Glc) and fructose (Fru)] and sucrose synthases (SuSys) [sucrose combines with uridine diphosphate (UDP) to generate fructose and UDP-glucose (UDP-Glc)] (Koch, 2004). These processes typically degrade sucrose *in vivo*, but the products of their reactions differ in important ways (Tymowska-Lalanne and Kreis, 1998; Winter and Huber, 2000). Invertases produce glucose instead of UDP-Glc, and thus also form twice as many Hexs. As an important transportation form of photosynthetic products, in common with fumaric acid, sorbitol, mannitol, and raffinose, Suc is moved from leaves to sink tissues through the phloem by sucrose transporters (SUTs) (Riesmeier et al., 1994; Patrick et al., 2013). These are clusters of transmembrane proteins that guide sugars across the membrane to implement the transport between phloem and source/sink cells by an apoplast upload and download pathway. Suc is loaded into the phloem by the respective sugar transporters, the sieve element/companion cell (SE/CC) complex or through plasmodesmata connecting the SE/CC complex. Suc is degraded by four different enzymatic mechanisms (Koch, 2004; Vandeputte and Delcour, 2004; Jiang et al., 2014). First, Suc is hydrolyzed into Hexs (Glc and Fru) by cell wall invertase (CWIN; EC 3.2.1.26) before entering the cytoplasm. Hexs are then transported into the cytoplasm by symporters. Secondly, Suc that is imported through plasmodesmata may be degraded by SuSy, producing UDP-Glc and Fru. Thirdly, Suc taken up the cytoplasm is hydrolyzed into Glc and Fru by a cytoplasmic invertase (CIN). Fourthly, cytosolic Suc may be carried into vacuoles for hydrolysis by vacuolar invertase (VIN). CWIN and VIN activities are limited by their respective inhibitors (INHs). The intracellular Hex is used for glycolysis and in the synthesis of sugar polymers. Additionally, the Hex level may also be sensed by the nucleus-localized hexokinase (HXK) or other proteins to regulate gene expression. Suc synthesis mainly involves a single pathway in which Hexs (Glc and Fru) are converted into hexose-6-phosphates by HXK (EC 2.7.1.1). Fru-6-phosphate and UDP-Glc are converted into Suc-phosphate (Suc-P) by Suc-P synthase (SPS), and then Suc-P is converted into Suc and phosphate by the action of Suc-P phosphatase. At the same time, a small amount of trehalose (T) is synthesized, which is a disaccharide and present in plants at low micromolar concentrations, suggesting a regulatory rather than a metabolic role (Paul et al., 2008); using Glc-6-phosphate and UDP-Glc as substrates, T-6-phosphate (T6P) synthase (TPS) first synthesizes T6P, which is then converted to T by T6P phosphatase (TPP). Finally, T is hydrolyzed by trehalase (Tl) into two molecules of Glc (Figure 1; Cabib and Leloir, 1958; Koch, 2004; O'Hara et al., 2013; Li et al., 2014; Ruan, 2014).

Reports on bulbous plants, such as *Gladiolus hybridus* (He et al., 2008), *Tulipa gesneriana* (Yu et al., 2009), and *Lilium* (Zheng et al., 2012; Li et al., 2014), have focused on starch or Suc metabolism. These studies have indicated that starch or Suc metabolism play an important role in the formation and development of bulbs. As with other bulbs, Suc metabolism is crucial in the development of onion (*A. cepa* L.) bulbs. In the study reported here, RNA-Seq, which is a powerful approach for detecting both differentially expressed genes (DEGs) and novel expressed genes over a broad dynamic range (Blencowe et al., 2009; Wang et al., 2009), has been used to elucidate Suc metabolism in onion, with the following objectives: (i) to identify DEGs involved in the formation and development of onion bulbs; and (ii) to screen the critical genes that are responsible for the changes in Suc, Glc, and Fru metabolism during the swelling of onion bulbs.

## Materials and methods

### Plant material and sample collection

The Utah Yellow Sweet Spain cultivar “Y1351” was used in this study. Fresh, undamaged onion (*A. cepa* L) bulbs were obtained from Beijing Academy of Agriculture and Forestry Vegetable Research Center. The bulbs were monitored until they formed a definite shape (bulbous) and size (7-cm diameter, with weights of 260–300 g), and samples were collected at three time points for cDNA library construction. The number of samples collected was ~10 on the 15th day after swelling (DAS) of bulb (2-cm diameter and 3–4 g weight), ~6 on the 30th DAS (5-cm diameter and 100–110 g weight), and ~3 on the 40th DAS (7-cm diameter and 260–300 gram), with three biological replicates per time point. Fresh samples were washed in running water to remove dirt and excess water was removed with blotting paper. Using a scalpel, 1–2 cm was excised from the top and the bottom of each bulb, and the remainder of the bulb was divided into three portions and used as the experimental material. For real-time quantitative PCR, material from bulbs was harvested at the three different time points, frozen immediately in liquid nitrogen and stored at −80°C prior to use.

### RNA isolation and cDNA library construction

Total RNA from each sample was isolated using TRIzol Reagent (Invitrogen, Carlsbad, CA, USA) following the manufacturer's instructions, with the modification that, to remove the supernatant fluid, precipitation with isovolumetric *iso*-propanol and high-salt solution (1.2 mol/L sodium chloride and 0.8 mol/L sodium citrate) was used instead of *iso*-propanol precipitation. The RNA concentration prior to gel electrophoresis was determined using a NanoDrop 2000 spectrophotometer (Thermo Fisher Scientific, USA); and to confirm the quality of RNA for further processing, the integrity of total RNA samples was determined using a RNA Nanodrop 6000 Assay Kit of the Bioanalyzer 2100 system (Agilent Technologies, CA, USA). The RIN (RNA integrity number) values of these samples were 9.3–9.6. The construction of the libraries and RNA-seq were performed by the Biomarker Biotechnology Corporation (Beijing, China).

The cDNA libraries from 15 DAS, 30 DAS, and 40 DAS samples were generated using an Illumina TruSeq™ RNA Sample Preparation Kit (Illumina, San Diego, CA, USA). Briefly, mRNA was enriched and purified from 3 μg of total RNA using oligo (dT)-attached magnetic beads and broken into short fragments using Illumina proprietary fragmentation buffer at 94°C for 15 min. Taking these cleaved mRNA fragments as templates, first-strand cDNA was synthesized using random hexamers and SuperScript II. Second-strand cDNA was then synthesized using DNA polymerase I, dNTPs, and RNaseH. These cDNA fragments were then subjected to an end-repair, poly(A) tailing was added, together with a ligation sequencing adapter, and the fragments were then purified and size-selected using AMPure XP beads. Next, PCR amplification was performed to enrich the purified cDNA template. Finally, the nine libraries were sequenced using an Illumina HisSeq™ 2500.

### *De novo* transcriptome assembly and annotation

Using the HisSeq™ 2500, transcriptome sequencing generated 100-bp pair-end (PE) raw reads. For further analysis, the raw reads were filtered by removing the adaptor reads and low-quality reads or unknown nucleotides. The rest of the clean reads of sufficiently high quality were generated and then assembled using the Trinity platform (http://trinityrnaseq.sourceforge.net/) for transcriptome assembly without a reference genome, with the parameters of “K-mer = 25, group pairs distance = 300,” and with other parameters set to default values (Grabherr et al., 2011). For each library, short reads were first assembled into longer contigs based on their overlap regions. The contigs were subjected to clustering to form the components. Different contigs from other transcripts and their distance were then recognized by the approach of De Bruijn graph assembly, and by mapping clean reads back to the corresponding contigs based on pair-end information, and thus the transcripts were produced. Finally, the potential transcript sequences were clustered using the TGI Clustering tool in order to obtain uni-transcripts (Pertea et al., 2003).

The unigene sequences were searched against the following public databases: Non-redundant protein (NR) database (Deng et al., 2006), Swiss-Prot (Apweiler et al., 2004) Gene Ontology (GO) database (Ashburner et al., 2000), Clusters of Orthologous Groups (COG) database (Tatusov et al., 2000), and Kyoto Encyclopedia of Genes and Genomes pathway (KEGG, Kanehisa et al., 2004). If the results from different database searches conflicted, then the order of priority was as follows: NR, KEGG, Swiss-Prot, GO, and COG. The threshold values for NR and Swiss-Prot were cut-off *e*-values of 10^−5^ and for COG it was a cut-off *e*-value of 10^−3^. The Blast2GO program (Conesa et al., 2005) was used to obtain GO annotation of the unigenes. WEGO and Top GO software (https://www.bioconductor.org/packages/2.12/bioc/html/topGO.html) was then used to perform GO functional classification and enrichment analysis of all the unigenes, in order to view the distribution of gene functions.

### Differential gene expression analysis

To compare the abundantly expressed genes in different samples, Bowite software (Langmead et al., 2009) was used to align between unigenes and reads obtained from each sample, and then associated with RSEM (Li and Colin, 2011) to estimate the expression of unigenes. Transcript count information for sequences corresponding to each unigene was calculated and normalized to the fragments per kilobase of transcript per million mapped reads (FPKM; Trapnell et al., 2010) values, in order to eliminate the influence of gene length and sequencing errors on the gene expression calculation. Significant DEGs were screened using DESeq software (Anders and Huber, 2010). The corrected *P*-values from this method accounted for multiple tests used the key factor, which was false discovery rate (FDR). FDR < 0.01 and |log_2_ (fold change)| > 1 or < −1 were set as the thresholds for differential gene expression. Fold changes in the expression levels between samples were used as the criteria in the screening process.

### Suc, Glc, and Fru assays

The levels of Suc, Glc, and Fru in bulbs at the three different developmental stages were determined by high-performance liquid chromatography (HPLC). Briefly, 1.0 g fresh onion bulb tissue was lyophilized and extracted in 40.0 mL distilled water by sonication for 30 min. The sonicated solution was then centrifuged at 13,000 × *g* for 5 min and filtered through a 0.45 μm Millipore filter prior to analysis. The sugar contents at the three different bulb developmental stages were determined in three independent experiments and carried out using three biological replicates.

### Quantitative real-time PCR analysis

To confirm RNA-seq results (DEGs selected) and to determine the roles of key enzymes involved in sucrose, glucose and fructose metabolism, quantitative real-time PCR (qRT-PCR) was performed using SYBR® green (TaKaRa, Dalian, China). The 1st-strand cDNA was synthesized using a PrimeScript II 1st Strand cDNA Synthesis Kit (Takara, Dalian, China). β*-Actin* was used as an internal reference gene. SYBR® green primers for quantitative real-time PCR were designed used Primer Premier 5.0 software. Quantitative real-time PCR was performed on a Roche Light Cycler 480 system (Bio-Rad, USA). The reaction mixture total volume of 20 μL contained 10 μL of 2 × SuperReal PreMix Plus, 2 μL of cDNA mix, 0.6 μL of each primer, and 6.8 μL of RNase-free double-distilled H_2_O. The program for RT-PCR was 95°C for 15 min, followed by 40 cycles of 95°C for 10 s, 60°C for 20 s, and 72°C for 20 s. A melting curve analysis was performed at the end of each PCR reaction at 95°C for 5 s, 65°C for 60 s, and 97°C continuously, prior to termination at 40°C for 30 s. Relative mRNA levels were calculated using the 2^−ΔΔCT^ method to normalize and calibrate gene expression levels relative to the internal reference β*-actin* (Livak and Schmittgen, 2001). Genes and primer sequences can be found in Table S1.

## Results

### Collection of bulbs

The transcriptome of onion (*A. cepa* L.) cultivar “Y1351” bulbs was evaluated during development, with reference to characteristic physiological and morphological changes. The first developmental stage, at ~15 DAS, has elliptical-shaped bulbs; the second stage, at ~30 DAS, has suborbicular-shaped bulbs; and the third stage, at 40 DAS, has bulbous-shaped bulbs and is approaching maturity (Figures S1A–C).

### RNA-seq analysis

A total of 72.53 million clean reads from the samples were obtained following quality assessment and data filtering. The GC% of sequenced data from the three libraries was ~45%, and the percentage of reads with an average quality score >30 was >85%. This indicated that the accuracy and quality of the sequencing data were sufficient for further analysis. The general sequencing statistics are shown in Table S2. The distribution of the expression of the unigenes was similar in the three different samples, which indicated that there was no bias in the process of constructing the cDNA libraries (Figure S2).

Using Trinity, the reads were assembled into 79,376 unigenes with a mean length of 678 bp. The median contig (N50) length was 1093 bp and 28,724 unigenes had sequences of 200–300-bp in length (Figure S3).

### Gene annotation and function classification

**Figure 2:**
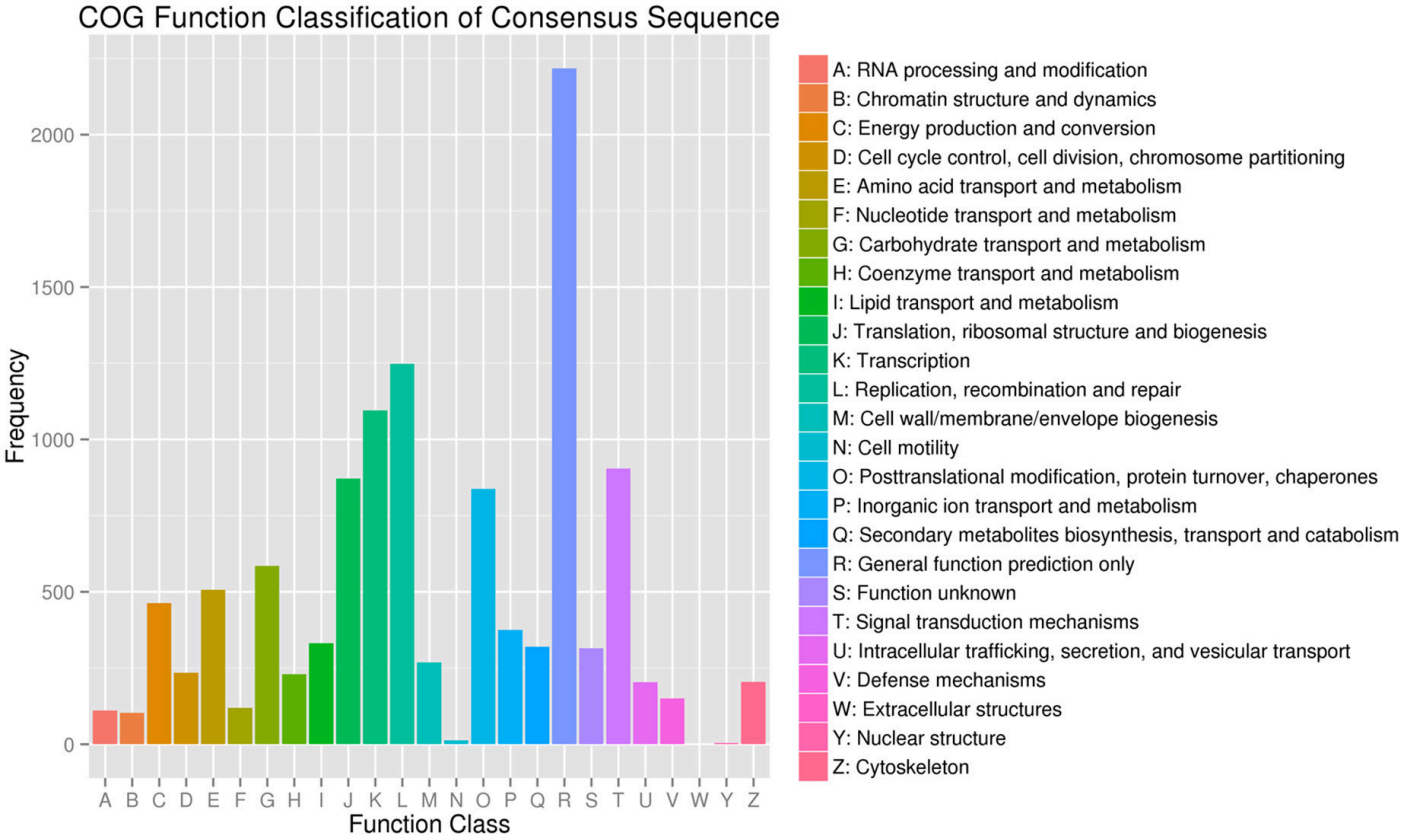
**Histogram of clusters of orthologous groups (COG) classification**. The 8288 unigenes were grouped into 25 COG categories.

The BLASTX algorithm was used to query the assembled sequences against Nr, Swiss-Prot, GO, COG, and KEGG databases, resulting in 27,955 (35.22%) perfectly annotated unigenes. Of these, 27,699 (99.08%) of the annotated unigenes were matched to the Nr database, while the smallest number of annotated unigenes was found in the KEGG database (Table S3). To further appraise the completeness of the RNA-seq data, COG classifications were performed with the annotated gene sequences. In total, 8288 unigenes were annotated according to 11,713 functions in 25 COG categories (Figure 2). Among them, “general function prediction only” (2217, 18.93%) ranked highest. Moreover, “carbohydrate transport and metabolism” was represented by only 585 unigenes (i.e., 7% of the annotated COG).

**Figure 3:**
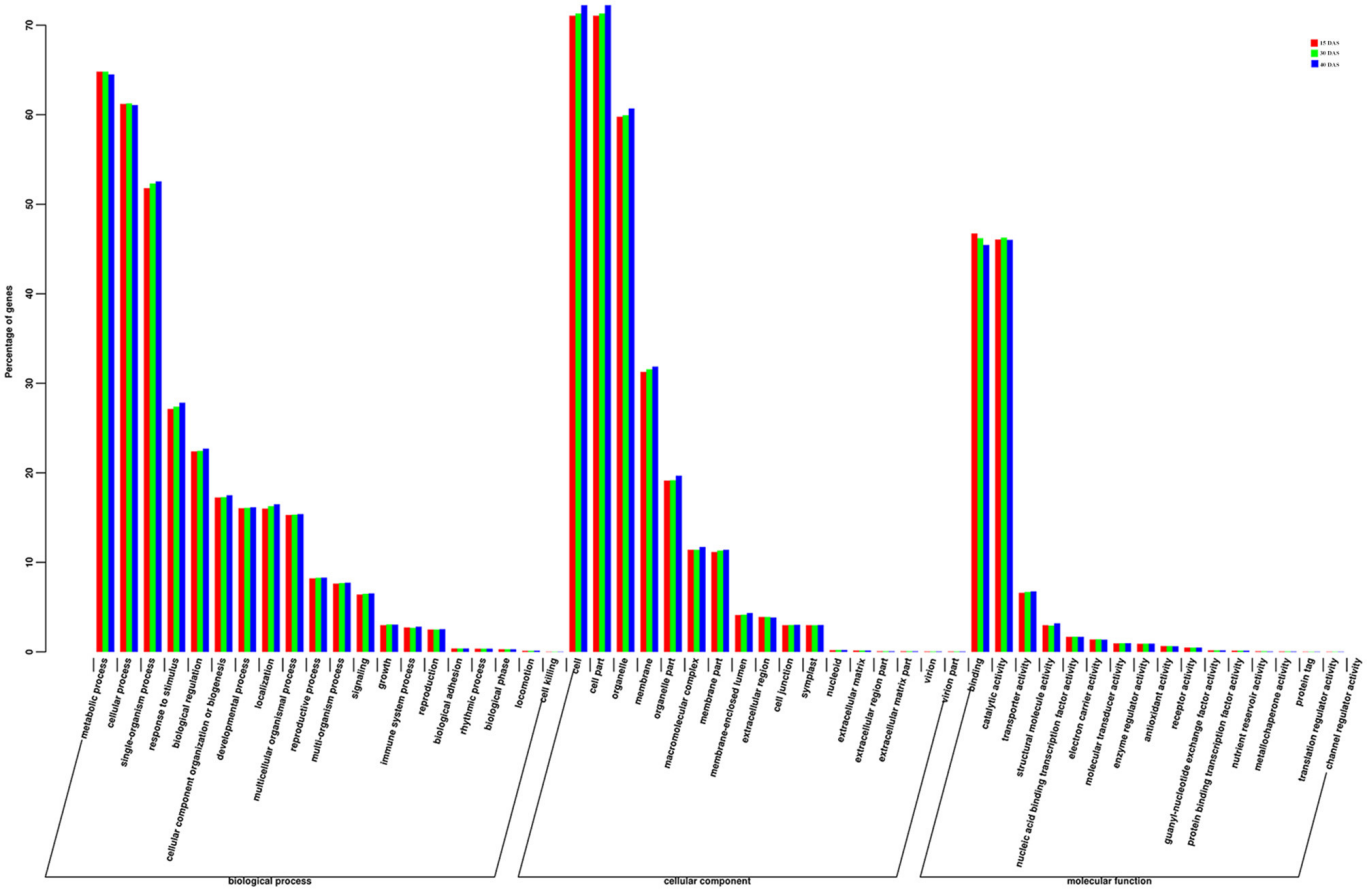
**Histogram of gene ontology (GO) classification for unigenes derived from bulbs in three developmental stages**. The unigenes corresponded to three main categories: “biological process,” “cellular component,” and “molecular function.” The left-hand y-axes indicate the percentage of genes.

Unigenes that were successfully annotated to the GO database were classified into three principal categories for the three different stages of bulb development, comprising “cellular component,” “molecular function,” and “biological process,” which were further subdivided into 56 categories (Table S4; Figure 3). Of these, the categories that were most represented in the “biological process” principal category were “metabolic process” [15 DAS: 11,737 (64.82%); 30 DAS: 11,522 (64.81%); and 40 DAS: 10,979 (64.5%)], which was the largest group, followed by “cellular process” [15 DAS: 11,084 (61.21%); 30 DAS: 10,894 (61.27%); and 40 DAS: 10,396 (61.08%)] and “response to stimulus” [15 DAS: 4912 (27.13%); 30 DAS: 4869 (27.39%); and 40 DAS: 4737 (27.83%)]. Within the “cellular component” principal category, unigenes [15 DAS: 12,866 (24.56%); 30 DAS: 12,678 (24.54%); 40 DAS: 12,293 (24.52%)] belonged to the “cell” category. In the principal category of “molecular function,” the two categories most represented were “binding” [15 DAS: 8461 (46.73%); 30 DAS: 8216 (46.21%); and 40 DAS: 7734 (45.44%)], and “catalytic activity” [15 DAS: 8339 (46.05%); 30 DAS: 8226 (46.27%); and 40 DAS: 7831 (46.01%)].

### Pathway enrichment analysis of DEGs

To identify the active pathways in the expansion stage of the onion bulb, the unigenes obtained were mapped to the canonical reference pathways in the KEGG database. KEGG pathway enrichment analysis was performed to categorize the biological functions of DEGs. Specific enrichment of genes was obtained for 95 pathways at 15 DAS vs. 30 DAS, for 79 pathways at 15 DAS vs. 40 DAS and for 86 pathways at 30 DAS vs. 40 DAS. Between library pairs, a total of 6113 unigenes were annotated in 120 KEGG pathways. The top 20 enriched pathways are listed in Table 1. “Ribosome” was the most common term and contained 401 (6.56%) DEGs, followed by “oxidative phosphorylation” (235, 3.84%), “protein processing in endoplasmic reticulum” (226, 3.70%), “plant hormone signal transduction” (202, 3.30%), and “RNA transport” (186, 3.04%). Notably, the other pathways were mainly involved in secondary metabolism, energy metabolism, protein processing, and RNA transport and splicing. But “starch and sucrose metabolism” (147, 2.40%) constituted the primary metabolism pathway in the integrated library and might be more active during bulb swelling and development.

**Table 1:** **The top 20 enriched KEGG pathways**.

**Pathway**	**Number of DEGs (% of total)**	**ID**
Ribosome	401 (6.56%)	ko03010
Oxidative phosphorylation	235 (3.84%)	ko00190
Protein processing in endoplasmic reticulum	226 (3.70%)	ko04141
Plant hormone signal transduction	202 (3.30%)	ko04075
RNA transport	186 (3.04%)	ko03013
Spliceosome	177 (2.90%)	ko03040
Glycolysis/Gluconeogenesis	162 (2.65%)	ko00010
Purine metabolism	147 (2.40%)	ko00230
Starch and sucrose metabolism	147 (2.40%)	ko00500
Ubiquitin mediated proteolysis	145 (2.37%)	ko04120
Plant-pathogen interaction	130 (2.13%)	ko04626
Ribosome biogenesis in eukaryotes	127 (2.08%)	ko03008
Pyrimidine metabolism	124 (2.03%)	ko00240
Amino sugar and nucleotide sugar metabolism	124 (2.03%)	ko00520
RNA degradation	122 (2.00%)	ko03018
mRNA surveillance pathway	116 (1.90%)	ko03015
Phagosome	107 (1.75%)	ko04145
Cysteine and methionine metabolism	101 (1.65%)	ko00270
Endocytosis	99 (1.62%)	ko04144
Citrate cycle (TCA cycle)	96 (1.57%)	ko00020

### Gene expression during bulb development

**Figure 4:**
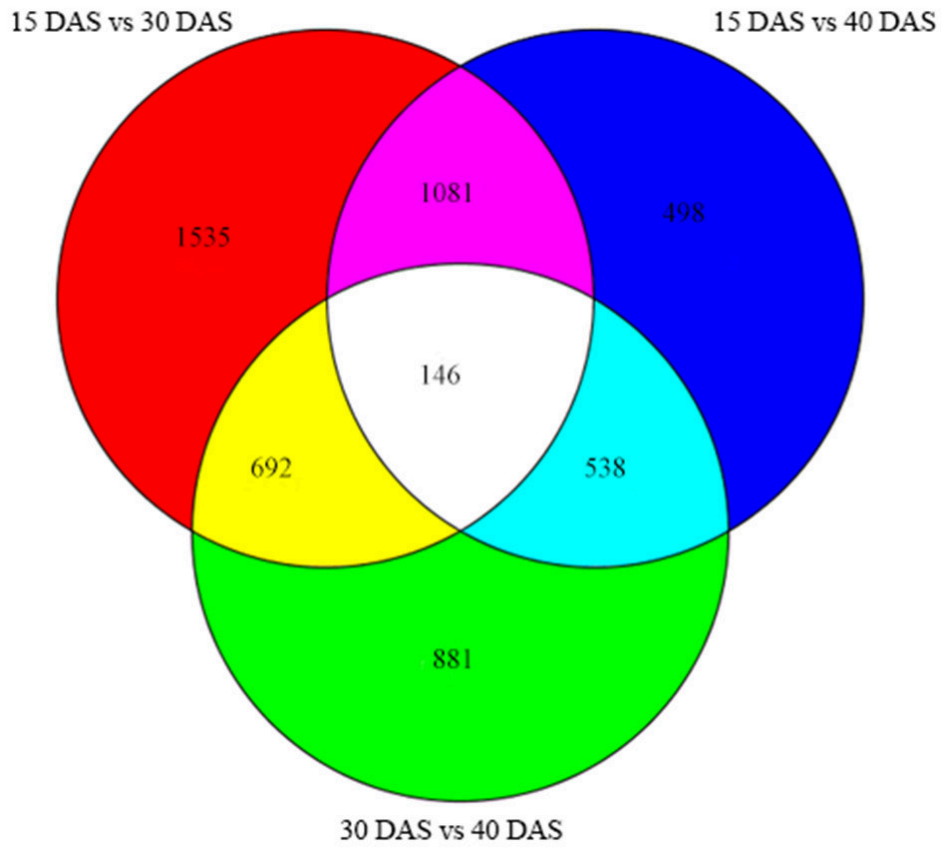
**Venn diagram showing genes differentially expressed in any two stages of onion bulb development**.

**Figure 5:**
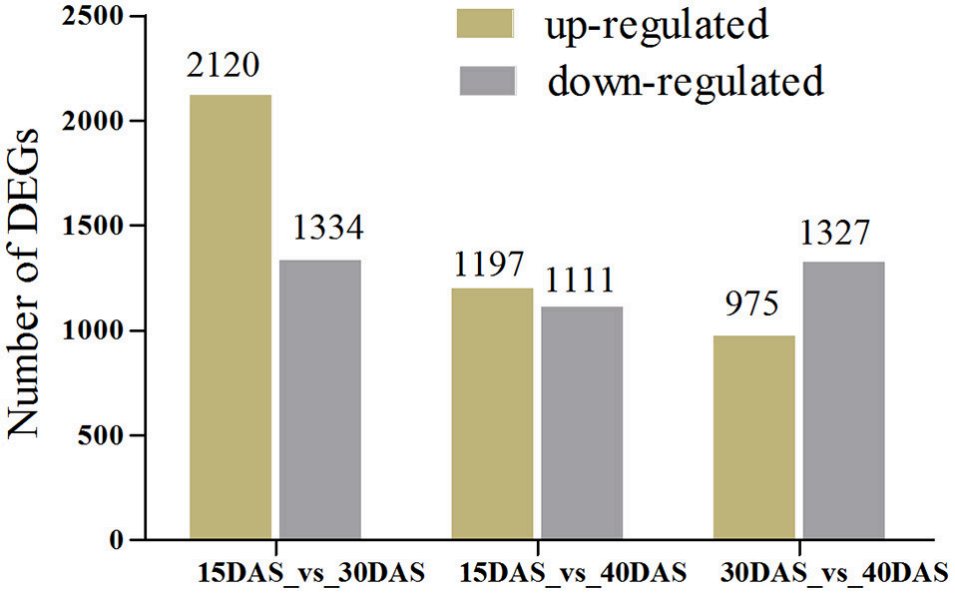
**Profile of the number of differentially expressed genes at different developmental stages**. The numbers of up- and down-regulated genes in comparisons of 15 DAS vs. 30 DAS, 15 DAS vs. 40 DAS, and 30 DAS vs. 40 DAS bulb samples are shown.

Setting a *P* < 0.01 and |log_2_ (fold change)| ≥ 1, DEGs involved in the development of bulbs were identified as up-regulated or down-regulated genes. As shown in the Venn diagram, a total of 146 DEGs were detected across all three pairs of developmental stages (Figure 4). The numbers of up- or down-regulated genes are shown in Figure 5. The numbers of up- or down-regulated genes changed markedly between 15 and 30 DAS, (2120 up-regulated vs. 1334 down-regulated genes), while the gene-expression pattern changed steadily between 15 and 40 DAS (1197 up-regulated vs. 1111 down-regulated genes). Between 30 and 40 DAS, 2308 DEGs were detected with 1197 up-regulated and 1111 down-regulated genes (Figure 5; Table S5).

### Determination of levels of sugars

**Figure 6:**
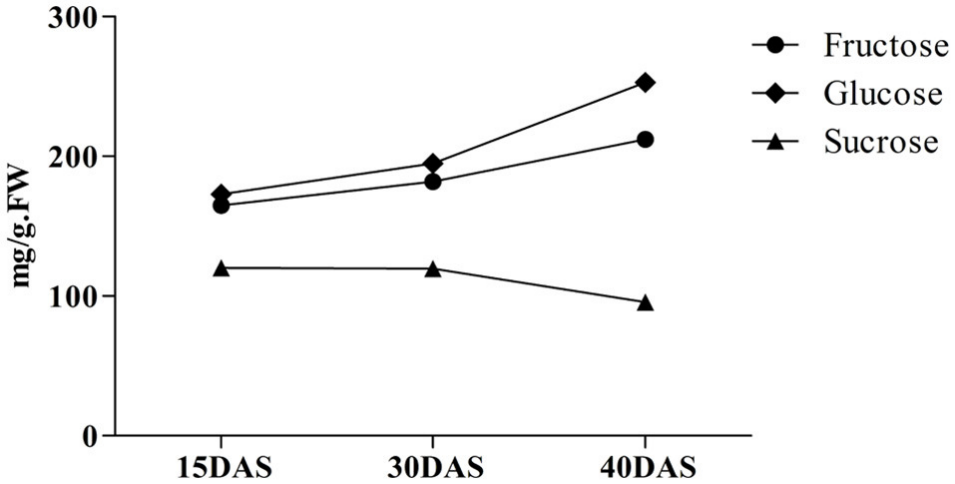
**Changes in the contents of Suc, Fru, and Glc at three different stages of onion bulb development**.

To investigate the accumulation and metabolism of sugars in swelling onion bulbs, the contents of Fru, Glc, and Suc at the three different developmental stages were measured by HPLC (Figure 6). At 15 DAS, the Glc and Fru contents were 172.93 mg/g fresh weight (fw) and 164 mg/g fw, respectively. The Suc content at this stage was at its maximum value, 120.22 mg/g fw. Interestingly, the Glc content (from 194.92 to 252.87 mg/g fw) in bulbs was higher than that (from 181.87 to 212.21 mg/g fw) of Fru and increased quickly from 30 to 40 DAS. At the same time, the Suc content decreased gradually from 15 DAS to reach 119.51 mg/g fw at 30 DAS and 95.63 mg/g fw at 40 DAS. This indicated that, after 30 DAS, bulbs started swelling rapidly, entering the rapid expansion period. After 40 DAS, the bulb had become essentially fully swollen and the Suc, Glc, and Fru contents barely changed (Grzelak et al., 2009).

### Critical genes involved in suc metabolism in onion bulb swelling

To gain a deeper insight into Suc metabolism during onion bulb swelling, a list of 147 differentially regulated starch and Suc metabolism genes was assembled by combining data from the literature and a keyword search in the onion RNA-seq annotation (Table S6). In the light of the changes in Fru, Glc, and Suc levels, we focused on six DEGs which were assumed to play important roles in Suc metabolism during the early stages of onion bulb swelling.

### Verification of gene expression by qRT-PCR

**Figure 7:**
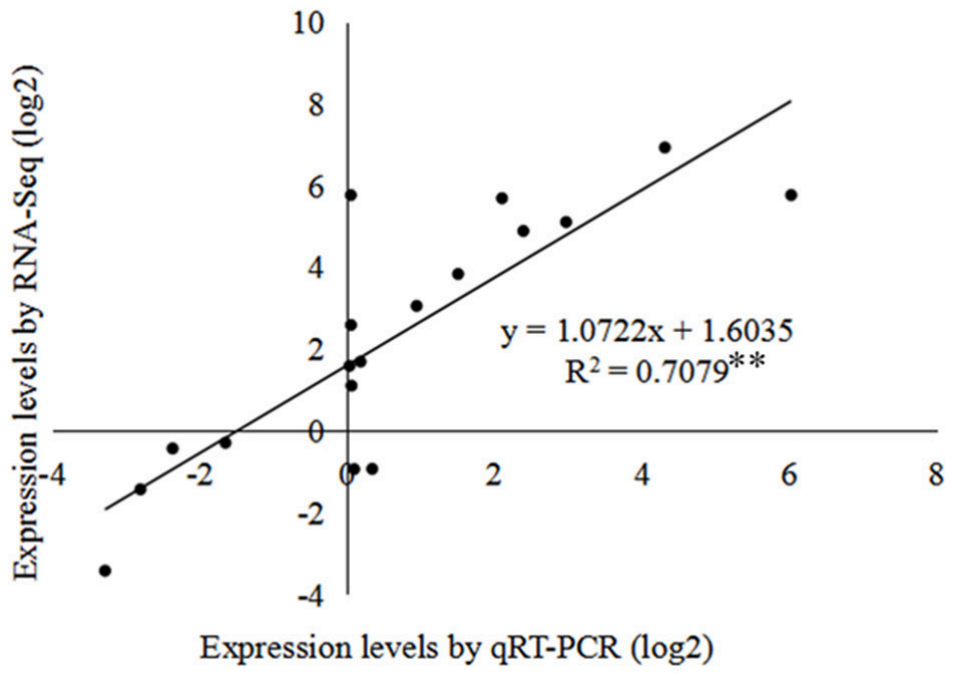
**Coefficient analysis of gene expression levels determined from RNA-seq and qRT-PCR data**. The qRT-PCR log_2_ values (x-axis) were plotted against onion bulb developmental stages (y-axis). ^**^Indicates a significant difference at *p* ≤ 0.01.

To confirm the RNA-seq results, six DEGs involved in Suc metabolism were selected for qRT-PCR analysis, comprising *CWIN, SUT, SuSy* (*SuSy1, SuSy2*), and *INV* (*INV1, INV2*). The results of the agarose gel electrophoresis indicated that all six primer pairs amplified a single band (Figure S4). The expression levels of the selected DEGs revealed by qRT-PCR were generally consistent with those from the RNA-seq analysis at the three developmental stages. Linear regression was [(qRT-PCR value) = a (RNA-Seq value) + b] and the correlation coefficient (*R*^2^) was 0.7079^**^ (Figure 7), which indicated that the results of the RNA-Seq analysis showed a high degree of correlation with those of qRT-PCR. The qRT-PCR values and FPKM values for the six genes are shown in Table S7.

**Figure 8:**
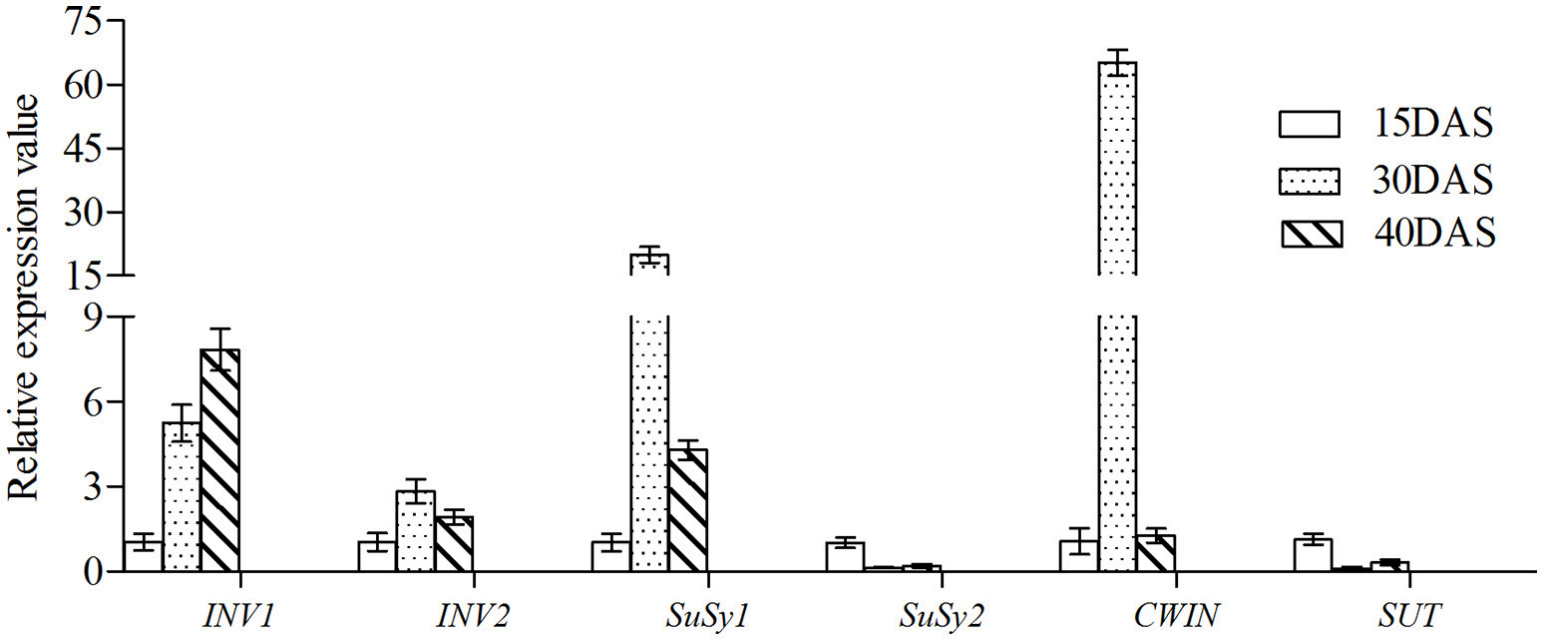
**Relative expression levels of six DEGs involved in Suc metabolism at three different bulb developmental stages**. The expression levels on the y-axis are relative to β*-actin* protein as control. Data are mean values of three biological replicates and error bars represent standard deviations.

The expression levels of the *SuSy2* and *SUT* genes were highest at 15 DAS, and then decreased gradually with time, which was consistent with the decline in Suc content after 15 DAS, during which period the Glc and Fru contents increased. This would be consistent with cleavage of Suc by *INV* and *CWIN*, in accordance with the relatively higher expression of the corresponding genes at 30 DAS (Figures 6, 8). After 30 DAS, the Suc content sharply decreased, which was consistent with an increase in the expression of *INV1*, whereas the expression of *CWIN* and *SuSy1* declined (Figure 8).

## Discussion

### The transcriptome and bulb development

RNA-seq, a powerful and efficient approach for gene expression analysis at the genome level, is especially useful in the case of non-model plants for which genomic sequence information is lacking. In this study, a total of 5416 DEGs were determined for the three different developmental stages of onion bulbs. Among them, more genes were found to be differentially expressed between 15 and 30 DAS than between the other pairs of developmental stages, with 2120 up-regulated genes and 1334 down-regulated genes. Correspondingly, the genes (*CWIN, SuSy1*, and *INV1*) associated with bulbs rapidly expanding were highly expressed at 30 DAS. These temporal gene expression patterns showed that the 30 DAS stage was the most active and rapidly expanding stage of bulb development; however, it was not clear why more DEGs were detected at 30 DAS.

The transcript distributions in the GO categories present a clear compositional depiction of the categories of genes involved in bulb formation and development. Among them, the principal category “biological process” accounts for 52,392 unigenes (42.20%) and the principal category “cellular component” accounts for 40.74%. Within “biological process,” 22.69% belonged to the category “metabolic process,” consistent with the involvement of genes of Suc metabolism in bulb development. According to the COG classifications, the category “carbohydrate transport and metabolism” was prominent, indicating that the morphogenesis and growth of bulbs requires the participation of carbohydrates. Furthermore, in the KEGG pathways, “starch and sucrose metabolism” (147, 2.40%) constituted the primary metabolism pathway in the integrated library.

### Suc metabolism in various plants

Besides its involvement in bulb development, Suc metabolism is also vital to fruit development. In the flavedo of *Citrus paradise*, there are ~22 significant enzymes involved in the synthesis of the major sugars during fruit ripening (Patel et al., 2014). A physiological analysis revealed that *Solanum lycopersicum* inhibitor mediated Suc metabolism by regulating *CWIN* activity, leading to an increase in fruit sugar content (Zhang et al., 2015). During pineapple (*Ananas comosus*) fruit development, the large rise in Suc was accompanied by dramatic up-regulated changes in SPS, and the cycle of Suc breakdown in the cytosol of sink tissues could be mediated by *SuSy* and *INV* (Zhang et al., 2012). The development of other fruits, such as grapes (Xin et al., 2013; Jiang et al., 2014) and peach (*Amygdalus persica* L.) (Desnoues et al., 2013) involves Suc metabolism. Moreover, Suc metabolism is prominent during flower blooming in *Rosa chinensis* “Pallida” (Yan et al., 2014).

Suc metabolism is crucial in tuber vegetables (which, though not bulbs, are examples of subterranean organ formation). Potato tubers (*Solanum tuberosum* L.) regulate Suc and starch metabolism in response to short-term water deficit, and there is a cycle of Suc degradation and re-synthesis in tuber discs of potato (Geigenberger, 1993; Geigenberger et al., 1997; Ferreira et al., 2010). Carrots (*Daucus carota* L.) expressing antisense mRNA for SuSy show markedly reduced SuSy activity in taproots (Tang and Sturm, 1999), and similar observations have been made in sweet potato (*Ipomoea batatas* L) (Firon et al., 2013) and turnip (*Brassica rapa* L.) (Gupta et al., 2001). In the current study, RNA-seq revealed 147 differentially regulated sucrose metabolism genes (Table S6). In the KEGG pathway analysis, “starch and sucrose metabolism” constituted the primary metabolism pathway (Table 1). All of the studies in the literature support the view that Suc metabolism is essential for bulb development in bulbous ornamentals (Zheng et al., 2012).

### Gene expression during bulb development

SUTs play a central role, as they orchestrate sucrose allocation both intracellularly and at the whole plant level. Suc produced in mesophyll cells of leaves may be effluxed into the apoplasm of mesophyll or phloem parenchyma cells by SUTs or by sucrose/H^+^ antiport (Kühn and Grof, 2010). The expression of *SUT* genes has been found to be very high in young tissues, consistent with our present studies (Figure 8). CWINs are typically expressed in sinks in which phloem unloading or subsequent postphloem transport follows an apoplasmic pathway. For example, in young tomato fruit, CWIN is abundant in the placenta phloem parenchyma, where Suc is unloaded apoplasmically (Jin et al., 2009). Interestingly, there is little or only weak *CWIN* expression in the phloem of the tomato fruit pericarp, which exhibits symplasmic unloading early in development (Ruan and Patrick, 1995; Jin et al., 2009). These studies showed that apoplasmic and symplasmic pathways could operate simultaneously in different cellular sites within an organ, matched by high and low CWIN levels, respectively. In the present study, the expression level of *CWIN* was highest at 30 DAS (Figure 8), which was consistent with the subsequent decrease in the content of Suc and with the fact that the bulbs were undergoing rapid expansion.

SuSy can reversibly catalyze Suc breakdown, whereas INV catalyzes Suc breakdown irreversibly (Li et al., 2014). Nevertheless (despite its name), SuSy is generally deemed to be involved in Suc hydrolysis rather than in Suc synthesis (Angeles-Núñez and Tiessen, 2010). In our study, the expression levels of *SuSy2* and *SUT* genes were relatively higher at 15 DAS compared to 30 DAS, or 40 DAS, whereas *CWIN, INV1*, and *INV2* genes were expressed at relatively low levels (Figure 8). It is possible that the Suc content increased gradually before 15 DAS; in this case, Suc would be mainly involved in metabolism prior to 15 DAS, providing energy for bulb swelling and development, and also playing a crucial role given that Suc serves as an important signaling molecule in relation to cellular metabolic status (Smeekens and Hellmann, 2014). Suc metabolism controls the generation of the sugar signaling molecules Suc, Glc, and T6P, and then regulates plant developmental processes, including meristem activation, by modulating the expression levels of transcription factors (Ruan, 2014). Consistently, the expression of some SuSy isoforms has been found to be induced or enhanced under hypoxia or anoxia in *Arabidopsis* (Bieniawska et al., 2007), in maize roots (Chourey et al., 1998), and in potato tubers (Fu and Park, 1995). The presence of the simple disaccharide, T, which is the major disaccharide of various bacteria, algae, fungi, and insects and which generates two Glc molecules on hydrolysis (Patrick et al., 2013), might explain why the content of Glc was still higher than that of Fru (Figure 6). VINs have long been considered to play a major role in cell expansion through their osmotic effect, and their activity is high in many rapidly expanding tissues and in tissues that are accumulating sugar, such as elongating *Arabidopsis* roots, expanding tomato fruits, and carrot taproots (Klann et al., 1993; Tang et al., 1999; Sergeeva et al., 2006; Ruan et al., 2010). The pattern of gene expression in the current work indicated that Suc content was positively correlated with the expression of *SuSy* and *INV* (Schäfer et al., 2004; Uys et al., 2007), which would be consistent with bulb swelling and the development of a sweet taste on account of increasing Glc and Fru content.

## Conclusions

In this study, we used three different developmental stages of onion (*A. cepa* L.) bulb, without access to a reference genome, and conducted a comparative gene expression analysis at the transcriptional level using RNA-seq. A total of 5416 DEGs were determined across the three different stages; of these, according to the KEGG database, 147 DEGs participated in starch, and Suc metabolism. Taking into account the content of Suc, Glc, and Fru at the different developmental stages, six critical DEGs involved in Suc metabolism, comprising *CWIN, SUT, SuSy* (*SuSy1, SuSy2*), and *INV* (*INV1, INV2*), were selected. The expression levels of *SUT* and *SuSy2* were highest at the early stage (15 DAS), and were lowest at 30 DAS; in contrast, the expression levels of *CWIN* and *INV* (*INV1, INV2*) increased. The findings were consistent with a decrease in the content of Suc and increases in the levels of Fru and Glc prior to 30 DAS. The expression levels of *CWIN, SuSy1*, and *INV2* were all maximal at 30 DAS, after which the content of Suc decreased, consistent with the onset of the rapid expansion period. After 30 DAS, the contents of Fru and Glc and the expression level of *INV1* sharply increased and reached maximum values at 40 DAS. These results suggest that programmed gene expression, together with changes in the contents of Suc, Fru, and Glc, are vital to bulb swelling. The findings will be of value in improving our understanding of the molecular mechanisms involved in bulb swelling and development in *Allium* spp. and closely related plants.

## Author contributions

CZ analyzed the data, performed the experiments, and drafted the manuscript. HZ participated in the data analysis and helped to draft the manuscript. ZZ helped to analyze the data. BL and ZC contributed analysis tools and helped to draft the manuscript. YL conceived the study, participated in its coordination, and helped to draft the manuscript. All of the authors have read and approved the final manuscript.

### Conflict of interest statement

The authors declare that the research was conducted in the absence of any commercial or financial relationships that could be construed as a potential conflict of interest.
