# Supplementary material for: Transcriptome Analysis of Sucrose Metabolism during Bulb Swelling and Development in Onion (Allium cepa L.)
Source: Front Plant Sci. 2016 Sep 22;7:1425. doi: 10.3389/fpls.2016.01425 (PMC5031786; doi:10.3389/fpls.2016.01425)

Supplementary Figure S1. Developmental stages of onion (*A. cepa* L.) 'Y13051' bulbs.
A: 15th day after swelling (DAS) of bulb; B: 30th DAS; C: 40th DAS.

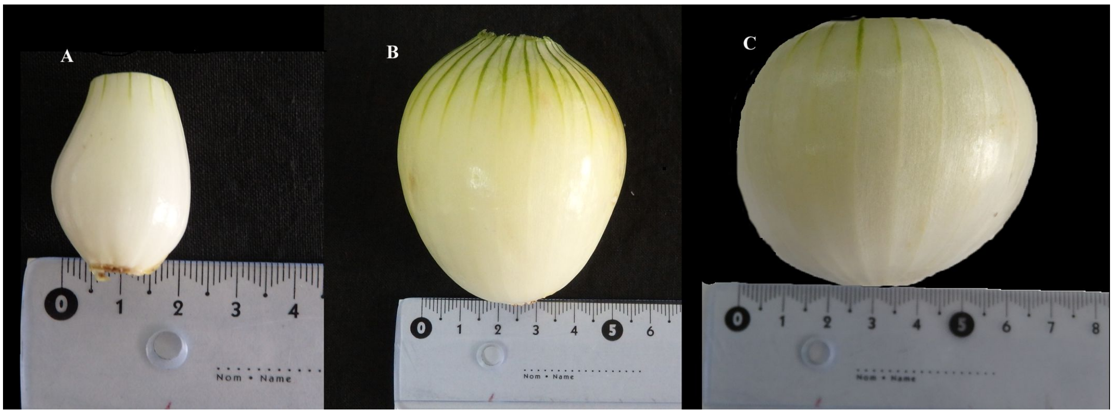

Supplement: Supplementary file 1 [file Image1.PDF]
