# Supplementary figures and images for: Transcriptome Analysis of Sucrose Metabolism during Bulb Swelling and Development in Onion (Allium cepa L.)
Source: Front Plant Sci. 2016 Sep 22;7:1425. doi: 10.3389/fpls.2016.01425 (PMC5031786; doi:10.3389/fpls.2016.01425)

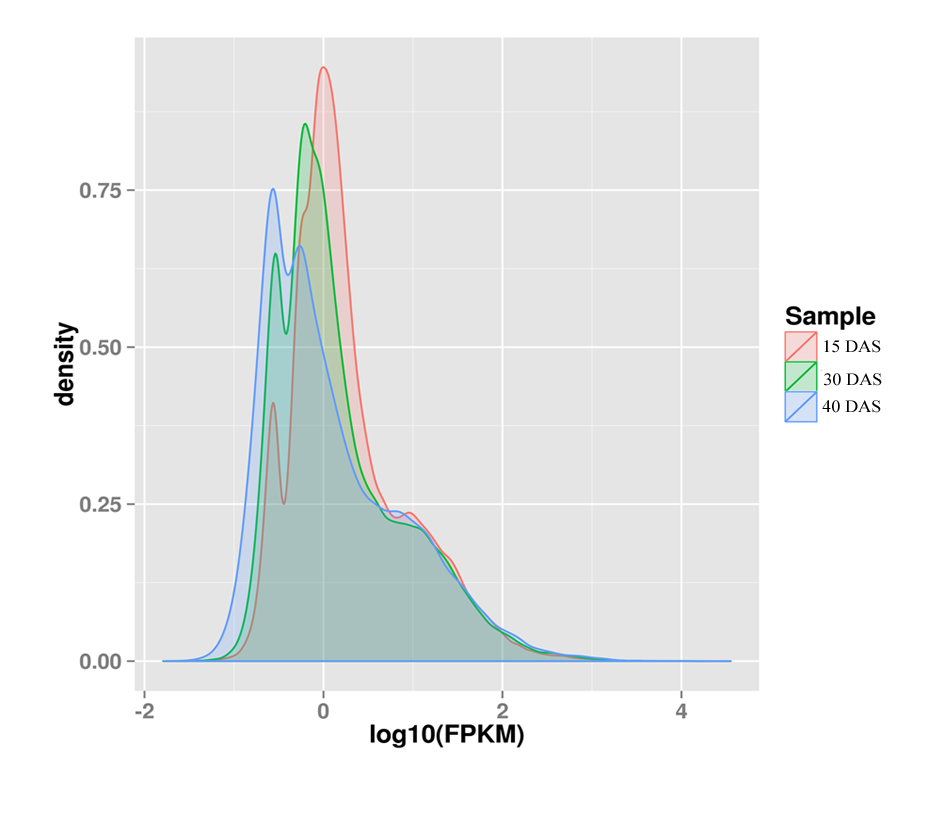

Supplement: Supplementary file 2 [file Image2.TIF]

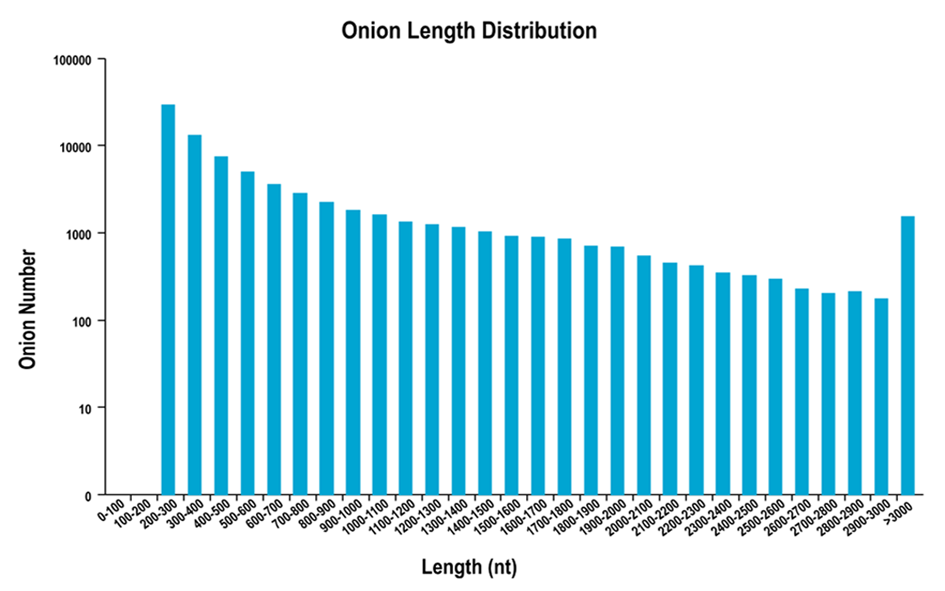

Supplement: Supplementary file 3 [file Image3.TIF]

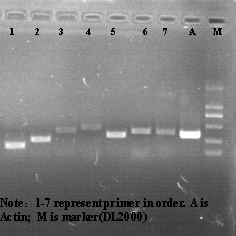

Supplement: Supplementary file 4 [file Image4.TIF]
