# Supplementary material for: Transcriptome Analysis of Sucrose Metabolism during Bulb Swelling and Development in Onion (Allium cepa L.)
Source: Front Plant Sci. 2016 Sep 22;7:1425. doi: 10.3389/fpls.2016.01425 (PMC5031786; doi:10.3389/fpls.2016.01425)
Supplement: Supplementary file 5 [file Table1.DOC]

***Supplementary Materials***

**Transcriptome Analysis of Sucrose Metabolism during** **Bulb Swelling and Development in Onion (*Allium cepa* L)**

**Chunsha Zhang^1^†, Hongwei Zhang ^1^†, Zongxiang Zhan^2^, Bingjiang Liu^3^, Zhentai Chen^4^, Yi Liang^1^***

**†**Chunsha Zhang and hongwei zhang contributed equally to this work

[***Correspondence**: Yi Liang, liangyi@nercv.org](mailto:*Correspondence: Yi Liang, liangyi@nercv.org)

**Supplementary Tables**

**Supplementary Table S1** The primers provided for quantitative real-time PCR

| **Gene/Unigene** | **Predict Function** | **Primer sequence** | |
| --- | --- | --- | --- |
| c8668.graph_c0 | Sucrose transporter | Forward | 5’-GCAACAAACAAGGATTGATAGC-3’ |
|  |  | Reverse | 5’-TGACATGGGTGGTTATG-3’ |
| c41126.graph_c0 | Sucrose synthase | Forward | 5’-GATGGGGAATTTGAGGAT-3’ |
|  |  | Reverse | 5’-TTGTATGGTGTTGACGC-3’ |
| c47870.graph_c0 | Sucrose synthase | Forward | 5’-ACGACTTGAAAGGGTG-3’ |
|  |  | Reverse | 5’-TAGGTGCTTGTTACGATA-3’ |
| c11587.graph_c0 | Cell wall invterase | Forward | 5’-GTGTTCAGACAATCCCAAGA-3’ |
|  |  | Reverse | 5’-GGCAGTCATAATCCCAGA-3’ |
| c35191.graph_c0 | Invertase | Forward | 5’-GCAGATTCAGGACGAT-3’ |
|  |  | Reverse | 5’-TAGGAAGATTTGTGGG-3’ |
| c16653.graph_c0 | Invertase | Forward | 5’-GCACCGCCAACATCTA-3’ |
|  |  | Reverse | 5’-AACCGACAGCGAAACT-3’ |
| *β-Actin* | -- | Forward | 5’-AGAGCAGTATTCCCAAGCATT-3’ |
|  |  | Reverse | 5’-ACACGGCCTGGATAGCAACAT-3’ |
