# Supplementary material for: Transcriptome Analysis of Sucrose Metabolism during Bulb Swelling and Development in Onion (Allium cepa L.)
Source: Front Plant Sci. 2016 Sep 22;7:1425. doi: 10.3389/fpls.2016.01425 (PMC5031786; doi:10.3389/fpls.2016.01425)
Supplement: Supplementary file 6 [file Table2.DOC]

***Supplementary Materials***

**Transcriptome Analysis of Sucrose Metabolism during** **Bulb Swelling and Development in Onion (*Allium cepa* L)**

**Chunsha Zhang^1^†, Hongwei Zhang ^1^†, Zongxiang Zhan^2^, Bingjiang Liu^3^, Zhentai Chen^4^, Yi Liang^1^***

**†**Chunsha Zhang and hongwei zhang contributed equally to this work

[***Correspondence**: Yi Liang, liangyi@nercv.org](mailto:*Correspondence: Yi Liang, liangyi@nercv.org)

| **Supplementary Table S2** Summary statistics of the three RNA-seq libraries created from onion bulbs at different days after swelling (DAS) | | | | | | |
| --- | --- | --- | --- | --- | --- | --- |
| Stage | Total Reads | GC Percentage | Q30 Percentage | Unique Mapping Reads | Multi Mapping Reads | Mapped Reads (%) |
| 15 DAS | 25452113 | 44.36% | 88.40% | 14975788 (71.44%) | 5987599 | 82.36% |
| 30 DAS | 23423040 | 45.14% | 89.65% | 14498340 (74.14%) | 5058184 | 83.49% |
| 40DAS | 23656951 | 45.71% | 88.63% | 15700163 (78.08%) | 4407976 | 85.00% |

**Supplementary Tables**
