# Supplementary material for: Transcriptome Analysis of Sucrose Metabolism during Bulb Swelling and Development in Onion (Allium cepa L.)
Source: Front Plant Sci. 2016 Sep 22;7:1425. doi: 10.3389/fpls.2016.01425 (PMC5031786; doi:10.3389/fpls.2016.01425)
Supplement: Supplementary file 7 [file Table3.DOC]

***Supplementary Materials***

**Transcriptome Analysis of Sucrose Metabolism during** **Bulb Swelling and Development in Onion (*Allium cepa* L)**

**Chunsha Zhang^1^†, Hongwei Zhang ^1^†, Zongxiang Zhan^2^, Bingjiang Liu^3^, Zhentai Chen^4^, Yi Liang^1^***

**†**Chunsha Zhang and hongwei zhang contributed equally to this work

[***Correspondence**: Yi Liang, liangyi@nercv.org](mailto:*Correspondence: Yi Liang, liangyi@nercv.org)

**Supplementary Tables**

| **Supplementary Table S3** Statistical results of unigene annotation. | | |
| --- | --- | --- |
| Annotated databases | Number of unigenes | Percentage (%) |
| COG | 8288 | 29.65 |
| GO | 18753 | 67.08 |
| KEGG | 6113 | 21.87 |
| Swiss-Prot | 17942 | 64.18 |
| Nr | 27699 | 99.08 |
| All | 27955 | 100.00 |
